# Top-down and bottom-up effects and relationships with local environmental factors in the water frog–helminth systems in Latvia

**DOI:** 10.1038/s41598-023-35780-7

**Published:** 2023-05-27

**Authors:** Andris Čeirāns, Mihails Pupins, Muza Kirjusina, Evita Gravele, Ligita Mezaraupe, Oksana Nekrasova, Volodymyr Tytar, Oleksii Marushchak, Alberts Garkajs, Iurii Petrov, Arturs Skute, Jean-Yves Georges, Kathrin Theissinger

**Affiliations:** 1 Department of Ecology, Institute of Life Sciences and Technologies, Daugavpils University, Daugavpils, Latvia; 2 I. I. Schmalhausen Institute of Zoology of National Academy of Sciences of Ukraine, Kyiv, Ukraine; 3 LOEWE Center for Translational Biodiversity Genomics, TBG - Senckenberg Nature Research Institute, Frankfurt, Germany; 4 CNRS, IPHC UMR 7178, Université de Strasbourg, Strasbourg, France

**Keywords:** Community ecology, Ecological epidemiology, Freshwater ecology, Herpetology, Wetlands ecology

## Abstract

Semi-aquatic European water frogs (*Pelophylax* spp.) harbour rich helminth infra-communities, whose effects on host population size in nature are poorly known. To study top-down and bottom-up effects, we conducted calling male water frog counts and parasitological investigations of helminths in waterbodies from different regions of Latvia, supplemented by descriptions of waterbody features and surrounding land use data. We performed a series of generalized linear model and zero-inflated negative binomial regressions to determine the best predictors for frog relative population size and helminth infra-communities. The highest-ranked (by Akaike information criterion correction, AICc) model explaining the water frog population size contained only waterbody variables, followed by the model containing only land use within 500 m, while the model containing helminth predictors had the lowest rank. Regarding helminth infection responses, the relative importance of the water frog population size varied from being non-significant (abundances of larval plagiorchiids and nematodes) to having a similar weight to waterbody features (abundances of larval diplostomids). In abundances of adult plagiorchiids and nematodes the best predictor was the host specimen size. Environmental factors had both direct effects from the habitat features (e.g., waterbody characteristics on frogs and diplostomids) and indirect effects through parasite-host interactions (impacts of anthropogenic habitats on frogs and helminths). Our study suggests the presence of synergy between top-down and bottom-up effects in the water frog–helminth system that creates a mutual dependence of frog and helminth population sizes and helps to balance helminth infections at a level that does not cause over-exploitation of the host resource.

## Introduction

Top-down and bottom-up interactions in ecology refer to direct interactions between adjacent trophic levels, where parasites are placed higher but their hosts lower accordingly to the concept of biomass pyramid^1^. Amphibian helminths include monogenean, trematode, cestode, acanthocephalan, and nematomorph worms, which all are endomacroparasites characterized by relatively large size and lack of asexual replication in vertebrates; they often have complex life cycles with one or more intermediate hosts, and cause infection intensity-dependent pathologies^2^.

While top-down interactions in amphibian–helminth systems, manifested through parasite-induced pathologies, reduced host fitness and survival, have been widely studied^2^, experimental studies on the bottom-up interactions are few and have been focused on host immunological response to helminth infections^3^ and effects of host nutrition and chemical composition of the food^4^. Moreover, interpretation of the results of a correlational research on parasite-host interactions in wild populations can be a difficult task, because most of the parasite-host systems in nature are open systems, integrated into food webs and exposed to environmental factors, often resulting in cryptic and complex interactions strongly affected by other components of the ecosystem^5^. For instance, waterbody eutrophication may lead to the snail community shift towards the taxa that are intermediate hosts for *Ribeiroia* trematode, which cause amphibian limb malformations^6^, nitrogen-rich forest litter may increase trematode infection in tadpoles via density- and trait-mediated effects on the snail intermediate hosts^4^, but alterations of the bird community structure may change helminth community composition in amphibians^7^.

Semi-aquatic water frogs from the *Pelophylax* genus harbour rich helminth communities dominated by up to 19 trematode species of both larval and adult stages, and up to 9 nematode species^8,9^. In Northern Europe water frogs form a three species complex with two parent (*Pelophylax lessonae, P. ridibundus*) and one hybridogenic (*P. esculentus*) species; the latter has a morphologically and ecologically transitional profile and is typically found in mixed populations with one or both parent species^10,11^. Top-down and bottom-up effects in water frog–helminth systems are poorly studied in nature. Despite numerous studies describing helminth infra-community composition and infection levels in European *Pelophylax* frogs, only a few refer to ecological factors, such as habitats^12,13^, host size^8,14,15^, or both^9^. Laboratory surveys have shown that helminth infections may have a detrimental effect on the water frog body condition. For example, acanthocephalans can cause severe pathological changes in the small intestine^16^ and the metacercaria of the diplostomid trematode *Strigea robusta* can cause polydactyly and other manifestations of Rostand’s anomaly P^17^. However, there are no fieldwork studies linking helminth infections with the frog population size estimates in nature and it is not clear how much they actually affect population welfare and sustainability (the presence of adverse effects can be suggested from a study showing that *S. robusta* may cause local population declines in European newts^18^). In turn, parasites depend on their hosts and a severe effect on their host populations would not fit an optimal virulence strategy^19^, parasites may potentially increase species coexistence^20^, but their community richness could be important driver for biodiversity and ecosystem productivity^21^.

**Figure 1:**
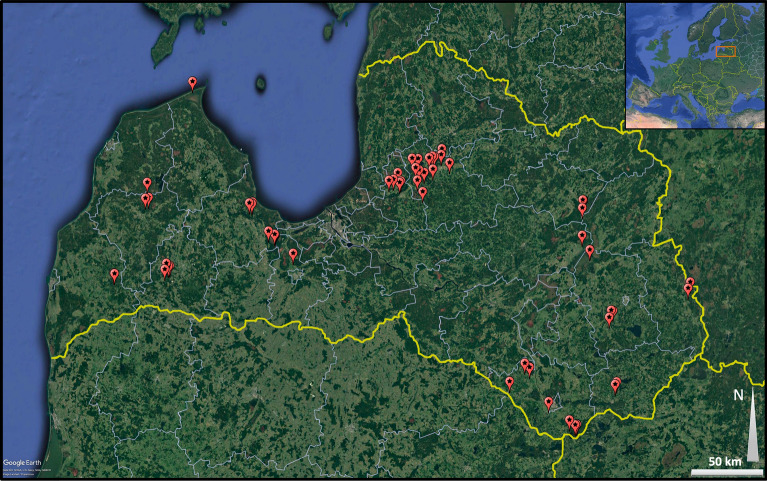
Location of study sites in Latvia; waterbodies that were sampled in 2018–2022 for the helminth infections in water frogs together with frog audible surveys (n = 63) denoted with the red pins. Maps were prepared in *Google Earth* v. 7.3 software using pin tool; attribution to the Google Earth seen in lower left corner; personalization of maps made accordingly to Google attribution guidelines published here: https://about.google/brand-resource-center/products-and-services/geo-guidelines/#required-attribution.

In Latvia, water frogs have been included in a state-wide monitoring programme since 2016, which is based on calling male counts rather than calling intensity metrics or presence/absence records traditionally used by other monitoring programmes^22^. This gave us an opportunity to collect relative amphibian population size data, which in this case is the number of calling males per waterbody, and plot it against parasitological survey results of the same population. During 2018–2022 we collected combined parasitological and water frog male calling data together with habitat descriptions from waterbodies across the whole country (Fig. 1). In the present study we asked and answered the following questions: (i) What has a larger effect on the water frog population size—helminth infections or habitat characteristics? (ii) Are there bottom-up effects from the water frog population size on helminth loads and their infra-community richness? iii) What is the effect of local environmental factors on the given parasite-host system?

## Methods

### Field works and sample collection

Data were collected in 2018–2022 at 63 waterbodies (Fig. 1). Their relative water frog population sizes were estimated during the male water frog calling surveys that were conducted at their maximum activity, in the first half of the warm nights of late May–early July. During these surveys the observer recorded the number of calling males (*Pelophylax lessone* and, tentatively, *P. esculentus*) over a period of at least five minutes (no maximum time limit). In cases with chorusing frogs, when counts were hindered by overlapping calls or banks were inaccessible and did not allow approaches close enough to distinguish individual calls, the number of calling males was recorded as an average between the minimum and maximum estimates by the observer^22^. Parasitological samples in these sites were taken in June–August of the same year. Water frog species identification may produce many errors when based only on morphological features^23^. A previous study showed a lack of substantial effect of water frog genetics on helminth communities^24^, therefore we did not separate water frog species in our study. At least three specimens of water frogs were collected at each waterbody (range 3–27, average ± SD 4.4 ± 3.4 specimens per site). Frogs were caught by a hand net, and each specimen was placed in a separate plastic box with water and aeration holes and transported to the lab for the parasitological survey. For each waterbody, the data form was filled and its essential features, such as waterbody and shoreline vegetation, were photographed. Field data forms included measurements of maximum depth, the composition of the bottom substrates (in categories as follows: 1 – sand, clay bottoms, 2 – partially mudded sand, clay, 3 – mud), visual estimations of percentage covers of submersed, floating, and emergent vegetation and descriptions of shoreline vegetation type.

### Parasitological survey

Parasitological investigations of collected frogs were carried out within 24 h after the samples were delivered to the laboratory. The frogs were first anaesthetized by the immersion in the buffered tricaine mesylate (MS-222) solution (2 g/L), and then euthanised by the pithing performed by FELASA Category C certified specialist^25^. Each frog specimen was measured, and a full standard parasitological investigation was carried out^26,27^, including examination of skin that was peeled off and rinsed in distilled water and all internal organs, body cavity, visceral membranes and limb musculature that were dissected, compressed between two slides and examined with a microscope. Encapsulated larval stages were released from surrounding tissues and analysed at × 100–400 magnification. Helminths were identified to the species level with the aid of essential references containing taxonomic keys and species descriptions, e.g.^28–30^, but in the data analyses we used higher ranked groups. Helminth species and life cycle stages were pooled into several main taxonomic and stage groups, of which four were present in 20 or more sites, and were used in further analyses. These were: a) larval (mesocercaria, metacercaria) stages of trematodes of the order Diplostomida; b) larval (metacercaria) stages of trematodes of the order Plagiorchiida; c) adult stages of trematodes of the order Plagiorchiida; d) adult stages of gastrointestinal Nematoda. Monogenea, Cestoda, and Acanthocephala were also present (represented by one species each), and they all contributed to the total helminth species richness in our study. Species lists of water frog helminth infra-communities from Latvia are given elsewhere^9^.

### Land use measurements

Proportions of various vegetation types on the shoreline or on the surrounding land were measured manually on digital orthophoto maps, using the Google Earth Pro software (Google LLC, Mountain View, California, USA) online tools. Manual measurements allowed us to verify the land use around each site in map sequences taken from several years. We measured several land cover features that were easily identifiable. On the waterbody shoreline these were proportions of the shoreline covered by wooded vegetation and dense tall reed stands (mainly *Phragmites australis* and *Scirpus* spp.; the latter were always verified by comparisons with the descriptions from field data forms and photos). Waterbody permanence was measured as the ratio of the smallest visible water table area to the maximum waterbody area in map sequences. We set 100 and 500 m wide belts around each shoreline, and within each belt we measured proportions of the following land use types: wooded vegetation (these were mostly forests, but also shrublands, gardens, etc.), mires (periodically or permanently wet areas with visibly distinct vegetation), agriculture (open areas with evidence of regular artificial vegetation removal), human settlements (buildings and home yards between buildings), and other waterbodies and watercourses. Other types of land uses and areas with unclear or questionable land use were omitted.

### Data sets

We used five variable groups (hereafter referred to as *ecological factors*) for our statistical models (full data set is given in the Supplement 1):

1) host variables (H), which included (i) water frog relative population size (calling males per waterbody, or *frogs* in our Results tables) and (ii) average frog size in our parasitology samples (or *host size*);

2) parasites (P) – abundances (*A*, or average number of worms per host for all samples including uninfected frogs) of (i) diplostomid larvae, (ii) plagiorchiid larvae, (iii) plagiorchiid adults, (iv) nematode adults, and (v) total helminth infra-community species richness (*S*, average per host) (species richness of separate helminth groups were also analysed, but had no statistically significant relationships with predictors and therefore were dropped from the present paper);

3) waterbody (W) – (i) area (ha), (ii) depth (m), (iii) permanence (ratio), (iv) the degree of mud in bottoms (category), coverages of (v) submersed, (vi) floating and (vii) emergent vegetations (%), and proportions of viii) woody vegetation and ix) reeds on the shoreline;

4), and 5) land uses within 100 m (L100) and 500 m (L500) belts around the waterbody, each containing proportions of coverages of (i) other waterbodies, (ii) mires, (iii) wooded vegetation (or *forest*), (iv) agriculture lands and (v) human settlements.

Parasite variables had no correlation with the number of sampled frogs per site (zero-inflated negative binomial (ZINB) regressions, p > 0.1 in all the cases). Average variance inflation factor (VIF) in the environmental predictor (W, L100, L500) data set was 3.55, indicating moderate collinearity (Table 1). However, their effects were separable and some collinearity did not hinder interpretations of the results. For instance, the highest VIF values were caused by a negative correlation between the *agriculture* and *forest* variables in the L500 group, which contrarily showed the same more often than the opposite direction of the relationship in our models.

**Table 1:** Values of variance inflation factor (VIF) for environmental variables in our regression analyses.

Variable	Variable group (ecological factor)	VIF	1/VIF
Agriculture500	L500	8.81	0.113
Forest500	L500	8.51	0.117
Forest100	L100	6.62	0.151
Mires100	L100	5.60	0.179
Mires500	L500	4.96	0.202
Agiculture100	L100	4.10	0.244
Settlements500	L500	4.09	0.245
Settlements100	L100	3.19	0.314
Shore woods	W	2.72	0.368
Floating	W	2.66	0.375
Area	W	2.18	0.460
Shore reeds	W	2.15	0.466
Emergent	W	2.10	0.476
Depth	W	1.94	0.516
Permanence	W	1.79	0.560
Waters500	L500	1.64	0.611
Mud	W	1.62	0.619
Waters100	L100	1.39	0.720
Submersed	W	1.37	0.728

### Data analyses

We performed two sets of regression analyses for our data set. In the first, frog relative population (or *frogs*) was the response variable and the ecological factors P, W, L100, and L500 were the predictors, and in the second, helminths (P group) were the response variables, but H, W, L100, and L500 were the predictors. A series of generalized linear model (GLM) regressions (Poisson identity) were performed for the analyses of frog population response, and zero-inflated negative binomial (ZINB) regressions (constant inflation option, logit model for characterizing zeros) for the helminth responses. ZINB is a recommended option to deal with overdispersed data sets with excessive zeroes like those in our helminth counts^31^. For each regression we used the backward elimination approach, when we stepwise removed predictors with smallest z-scores and *p* > 0.05 from the initial set of ecological factors, until we identified models with the lowest Akaike information criterion (AIC) values. A model was considered valid if it retained at least one variable from each ecological factor included in the initial set. Valid models were ranked according to their corrected Akaike information criterion (AICc). For statistically significant models, we used McFadden’s pseudo R^2^^32^ to compare goodness-of-fit between models with various response variables. All statistical analyses were performed on STATA 14.2 (StataCorp LLC, College Station, Texas, USA) with the Stata Technical Bulletin insertion ‘Scalar measures of fit for regression models’ (developed by J. Scott Long, Indiana University and Jeremy Freese, University of Wisconsin-Madison, USA).

### Ethical standards

The animals were euthanized in accordance with Directive 2010/63/EU of the European Parliament on the protection of animals used for scientific purposes and according to the guidelines of the Federation of European Laboratory Animal Science Associations (FELASA)^25^. The study was carried out in compliance with the ARRIVE guidelines version 2.0 (National Centre for the Replacement, Refinement & Reduction of Animals in Research, London, UK), all the investigation protocols were approved the Daugavpils University Ethical committee (decision Nr. 26/2). Special permission for the collecting, euthanasia and study for scientific purpose was approved in accordance with Latvian legislation by the Latvian authority—Nature Conservation Agency of Latvia (permission numbers 14/2018-E, 21/2019-E, 19/2020-E, 3/2021, and 10/2022).

## Results

Statistically significant relationships with other host-parasite-environment system components were found in both models explaining water frog population size (Table 2) and models explaining helminth abundances (*A*) and their species richness (*S*) (Table 3). Statistically significant models were found in 33% of all the possible ecological factor combinations when the water frog population was a response variable, in 73% combinations when it was adult plagiorchiid abundance (*A*), 60% when it was helminth infra-community richness (*S*), 27% when it was larval diplostomid (*A*), 20% when it was nematode (*A*), and 7% when it was larval plagiorchiid abundance (*A*). Akaike weights (w_i_(AICc)) indicated low to moderate relative likelihoods of the best models, varying from 0.378 in the best model for larval diplostomids, to 0.106 in larval plagiorchiids adults.

**Table 2:** GLM models for various combinations of ecological factors explaining water frog (*Pelophylax* sp.) relative population size, ranked by Akaike information criterion correction (AICc).

Ecological factor	AICc	ΔAICc	w_i_(AICc)	Predictors (z− score); *p < 0.05, ** *p* < 0.01, ***p < 0.001
W	4.955	0.000	0.258	Shore reeds (− 4.61)***, area (3.87)***
L500	5.330	0.375	0.214	Forests500 (− 3.86)***, settlements500 (− 3.76)***, agriculture500 (− 3.18)**
L100 + L500	5.544	0.589	0.192	Forest500 (− 4.70),*** agriculture500 (− 3.56)***, settlements500 (− 3.25)**, forest100 (2.62)**
W + L500	5.690	0.735	0.179	Forest500 (− 4.27)***, agriculture500 (− 3.88),*** area (3.62)***, settlements 500 (− 3.53)***, mud (2.11)*
P + L500	5.939	0.984	0.158	Forest500 (− 4.11)***, agriculture500 (− 3.64)***, settlements500 (− 3.64)***, S_helminths (− 2.72)**, Plagiorchiida adults (2.15)*

Ecological factors: P, parasite: W, waterbody and shoreline: L100, land use within 100 m distance: L500, land use within 500 m distance: ΔAICc, the difference in AIC score between the best model and the model being compared: w_i_(AICc), Akaike weight.

**Table 3:** ZINB models for various combinations of ecological factors explaining abundances (*A*) of various helminth groups and helminth infra-community species richness (*S*), ranked by Akaike information criterion correction (AICc ).

Ecological factor	AICc	ΔAICc	w_i_ (AICc)	Predictors (z-score); *p < 0.05, **p < 0.01, ***p < 0.001
Diplostomid larvae, A (n = 63, nonzero n = 31)				
W	4.831	0.000	0.378	Emergent (− 2.18)*
H + W + L500	5.541	0.710	0.265	Frogs (4.42)***, emergent (-3.59)***, mud (3.57)***, host size (3.00)**, waters500 (3.00)**
H + W	5.649	0.818	0.251	Frogs (4.03)***, emergent (− 3.68)***, permanence (− 2.38)*, host size (2.35)*, mud (2,16)*
W + L500	7.376	2.545	0.106	Waters500 (4,13)***, permanence (3.75)***, settlements500 (− 3,44)**, floating (3,19)**, depth (− 3,06)**, area (2,81)**, shore woods (− 2,17)*, agriculture500 (− 2.02)*
Plagiorchiid larvae, A (n = 63, nonzero n = 26)				
W	3.346	0.000	1.000	Area (− 2.36)*
Plagiorchiid adults, A (n = 63, nonzero n = 43)				
H	5.030	0.000	0.106	Host size (4.35)***
H + L500	5.114	0.084	0.102	Host size (4.32)***, waters500 (− 2.30)*
H + L100	5.125	0.095	0.101	Host size (4.24)***, mires100 (− 2.37)*
H + W	5.130	0.100	0.101	Host size (4.72)***, floating (1.97)*
L100	5.204	0.174	0.097	Mires100 (− 2.55)*
H + L100 + L500	5.277	0.247	0.094	Host size (4.21)***, mires100 (− 2.43)*, waters500 (− 2.35)*
L100 + L500	5.284	0.254	0.093	Mires100 (− 2.62)*, waters500 (− 2,49)*
W	5.315	0.285	0.092	Shore reeds (− 2.42)*, permanence ( 2.34)*
W + L100	5.740	0.711	0.074	Settlements100 (3.89)***, permanence(3.28)**, Submersed (2.34)*, agriculture100 (2.09)*
L500	5.809	0.779	0.072	Waters500 (− 2.55)*, agriculture500 (− 2.35)*, forest500 (− 2.20)*, mires500 (− 2.13)*
H + W + L500	5.884	0.854	0.069	Host size (5.31)***, mires500 (− 2.36)*, floating (2.20)*, frogs (2.01)*, waters500 (− 1.98)*
Nematoda, A (n = 63, nonzero n = 20)				
H	2.267	0.000	0.355	Host size (3,05)**
H + W	2.462	0.195	0.322	Host size (3.64)***, submersed (− 2.85)**, floating (2.45)*
W	2.464	0.197	0.322	Submersed (− 2.43)*, permanence (2.06)*
Helminths, S (n = 63, nonzero n = 53)				
H	3.480	0.000	0.129	Host size (3.88)***
H + L500	3.527	0.047	0.126	Host size (4.70)***, settlements500 (3.09)**
W	3.622	0.143	0.120	Emergent (− 2.30)*
H + W + L500	3.652	0.172	0.118	Host size (4.76)***, settlements500 (3.39)**, submersed (-2.66)**
H + W	3.716	0.236	0.115	Host size (4.20)***, submersed (− 2.50)*, floating (2.38)*
L100 + L500	3.780	0.300	0.111	Agriculture100 (2.40)*, agriculture500 (− 2.27)*
W + L500	3.907	0.427	0.104	Emergent (− 2.66)**, submersed (− 2.05)*, agriculture500 (− 2.04)*
H + L100 + L500	3.994	0.514	0.100	Host size (4.39)***, settlements500 (3.34)**, agriculture500 (− 2.21)*, agriculture100 (2.07)*
W + L100 + L500	4.492	1.013	0.077	agriculture500 (− 3.26)**, agriculture100 (3.2)**, emergent (− 2.43)*, shore woods (2.09)*, settlements500 (2.03)*

Ecological factors: H, host; W, waterbody and shoreline; L100, land use within 100 m distance; L500, land use within 500 m distance; ΔAICc the difference in AIC score between the best model and the model being compared; w_i_(AICc), Akaike weight.

In the model rankings by AICc, the most qualitative GLM model explaining water frog population size contained only the waterbody factor, and it was followed by the model containing land use within 500 m (L500) only. Models containing land use within 100 m or the parasite factor were significant only in combinations with the waterbody or L500 factor. The model containing parasite factor was on the bottom of the ranking by AICc.

In the larval diplostomid and the larval plagiorchiid abundances, AICc also ranked waterbody factor only as a top ZINB model, and in both cases they contained only one predictor (vegetation cover and area, respectively) and had large dispersion (as was indicated by their low z-scores and marginally significant p-values). In the larval plagiorchiids, effects from all the other ecological factors (H, L100, L500) or their combinations were not significant. In contrast, there were several statistically significant models for the combinations of ecological factors in diplostomids, with the second-ranked model indicating similar importance of host, waterbody, and land use (L500) factors, and frog population size having the highest z-score among all the predictors. In adult plagiorchiid (*A*) and nematode abundances (*A*), and in helminth infra-community species richness (*S*), the most important predictor was host size, with the waterbody factor ranked second or third. In adult plagiorchiids, three more ecological factors (W, L100, L500) had statistically significant combined models with the host factor. They were ranked second to fourth by AICc, and all produced similar weights (w_i_(AICc)) and low non-host predictor z-scores. As for helminth richness (*S*), the models with land use within 500 m were ranked higher than the models with land use within 100 m. Water frog population size was not a significant factor for nematodes and helminth species richness, and had only a weak, marginally significant positive relationship in the lowest-ranked model for adult plagiorchiids.

Goodness-of-fit varied between the groups of models for various parasite-host system members (Table 4). Models explaining nematode abundances had highest average fits to our data sets, followed by the helminth species richness and the water frog population size models. The lowest average McFadden’s pseudo R^2^ value was found in the larval plagiorchiid abundance (*A*) analyses, which produced only one statistically significant model.

**Table 4:** Comparison of goodness-of-fit of statistically significant models for different response variables, ranked by McFadden’s pseudo R^2^.

Response variable	Number of models	Regression type	logLIK, average	McPsR^2^, average	*P*, average
Nematoda, A	3	ZINB	13.56	0.122	0.0072
Helminth species richness, S	9	ZINB	18.30	0.083	0.0077
Water frog population	5	PR	25.77	0.081	< 0.0001
Diplostomid larvae, A	4	ZINB	19.34	0.065	0.0141
Plagiorchiid adults, A	11	ZINB	17.11	0.053	0.0058
Plagiorchiid larvae, A	1	ZINB	6.66	0.034	0.0099

*PR* Poisson regression, *ZINB* zero-inflated negative binomial regression, *logLIK* log likelihood ratio chi-square, *McPsR*^2^ McFadden’s pseudo R^2^, *P* likelihood ratio chi-square test.

## Discussion

### Top-down effects of helminths on water frog population size

**Figure 2:**
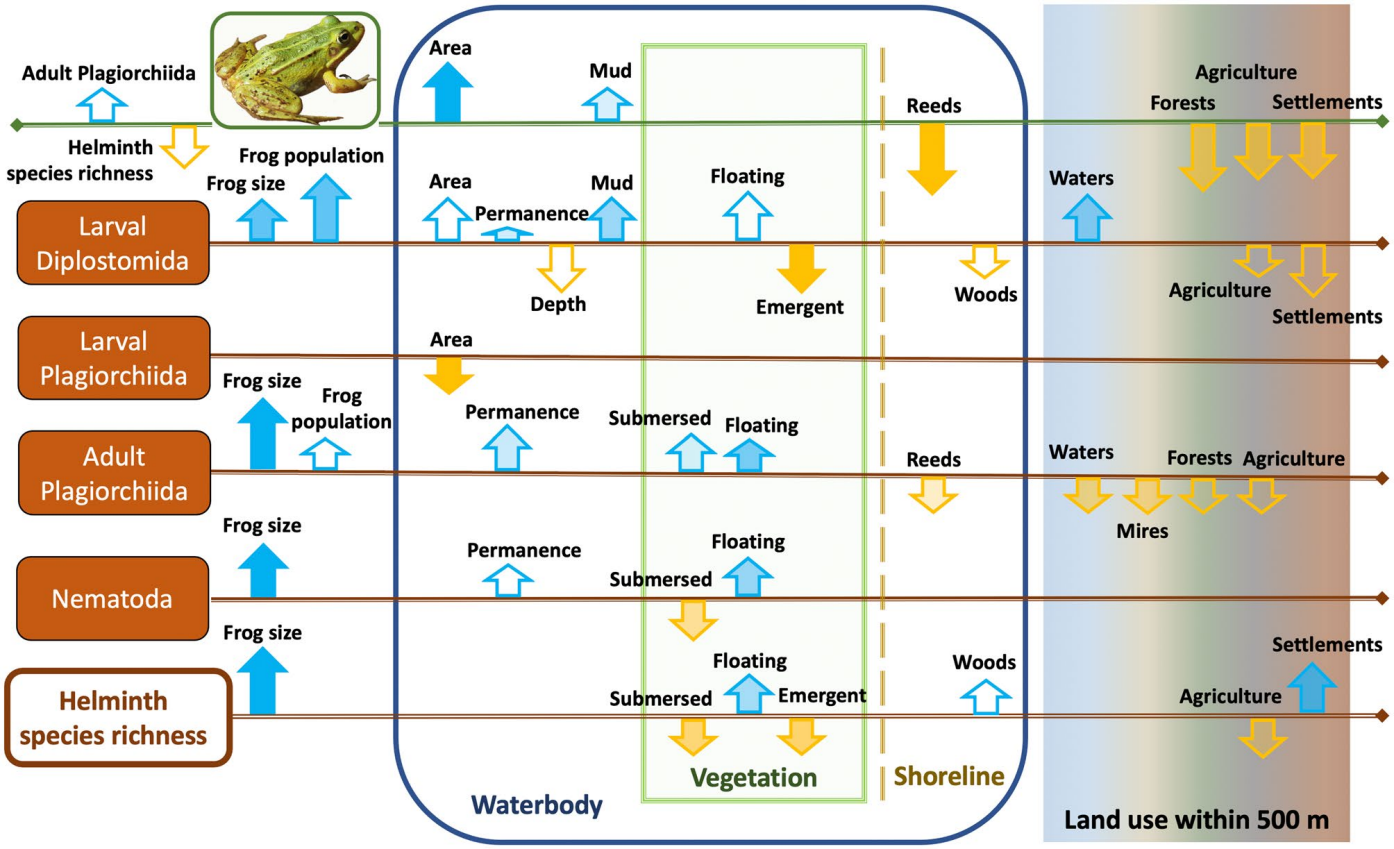
Ecological factors that affected water frog (*Pelophylax* sp.) population size (denoted with a picture of the water frog), abundances of their main helminth groups and their total helminth species richness in 63 waterbodies from Latvia, sampled in 2018–2022. Blue arrows indicate positive, orange arrows—negative relationships with a given factor in statistical models. Arrow heights are proportional to the average z-score in statistically significant models (generalized linear model regressions for the frog population size, and zero-inflated negative binomial regressions—for heminths), but the degree of their filling is proportional to the rank of the relative quality of their best model (Tables 2 and 3).

Top-down effects of parasites were markedly weaker than the effects from both waterbody characteristics and terrestrial habitats. Water frog population size had a strong relationship with waterbody area (positive) and shoreline features (dense reed stands on the shoreline were negative, probably because they limit access to basking places) (Fig. 2). Also, frog populations were smaller in areas with large proportions of both closed forests and anthropogenic habitats (agriculture, settlements). Habitats near waterbodies (100 m belt) only had an effect in combination with the land use in the larger area (500 m belt), when water frog populations in less forested areas tended to be larger when there were forested belts around the waterbody.

There were only two cases where helminth predictors correlated with the frog population size. The first case indicated a negative effect from the helminth infra-community richness, which is confirmed by increased amphibian mortality caused by this factor in a previous study^33^. The second case was a marginally significant positive frog population size relationship with adult plagiorchiid trematode infections. The latter relationship could be regarded as a covariation rather than the impact and was probably caused by the presence of abundant arthropod populations relevant to large frog populations, resulting in higher plagiorchiid transfer rates from arthropods to frogs, complemented by low pathogenicity of this helminth group^9,14^.

Helminth infections, especially in larval diplostomid trematode and gastrointestinal nematode infections, may have depressing effects on the water frog populations, when we consider both direct^17,18,34^ and indirect^9^ evidence from other studies. However, these groups showed no significant impact on the water frog population size in nature, and we generally agree with a statement made by Comas et al.^14^ that the host-parasite systems in water frogs seems to be evolved towards low levels of virulence and commensalism.

### Bottom-up effects from water frogs on helminths

The bottom-up effect from the host population size was observed only for larval diplostomid trematodes, which were more abundant in water frogs from larger populations, and this was an important factor with about the same rank as the most influential environmental factor (waterbody features). A marginally significant positive relationship between frog population size and adult plagiorchiids could be regarded as a covariation rather than the effect (see above).

There are several potential causes for higher larval diplostomid abundances in larger frog populations: (i) larger host populations increase host-parasite encounter rates and parasite transmission^35^; (ii) adult water frog individuals may step up as paratenic hosts due to cannibalism under the crowded conditions^29,36^; (iii) larger frog populations may have higher potential for transmissions to definitive hosts (birds and carnivores) that may tend to stay at sites with rich food resources^37^.

As for the adult worm abundances (both trematodes and nematodes) and helminth infra-community richness, the most important factor was the individual host specimen size. Harbouring of more parasites and species by a larger host is a well-known phenomenon caused by parasite accumulation and/or higher intake rates^8,14,38^. This has already been described for the water frog infra-communities of Latvia in our earlier work^9^. The effect from host size could formally be viewed as a bottom-up effect, but it could be also viewed as a preference for the host’s certain ontogenetic stages by a parasite. Hence, if we skip host size, the bottom-up effect on adult worm infections and helminth infra-community richness in our study was absent.

### Effect of environmental factors on helminths

**Figure 3:**
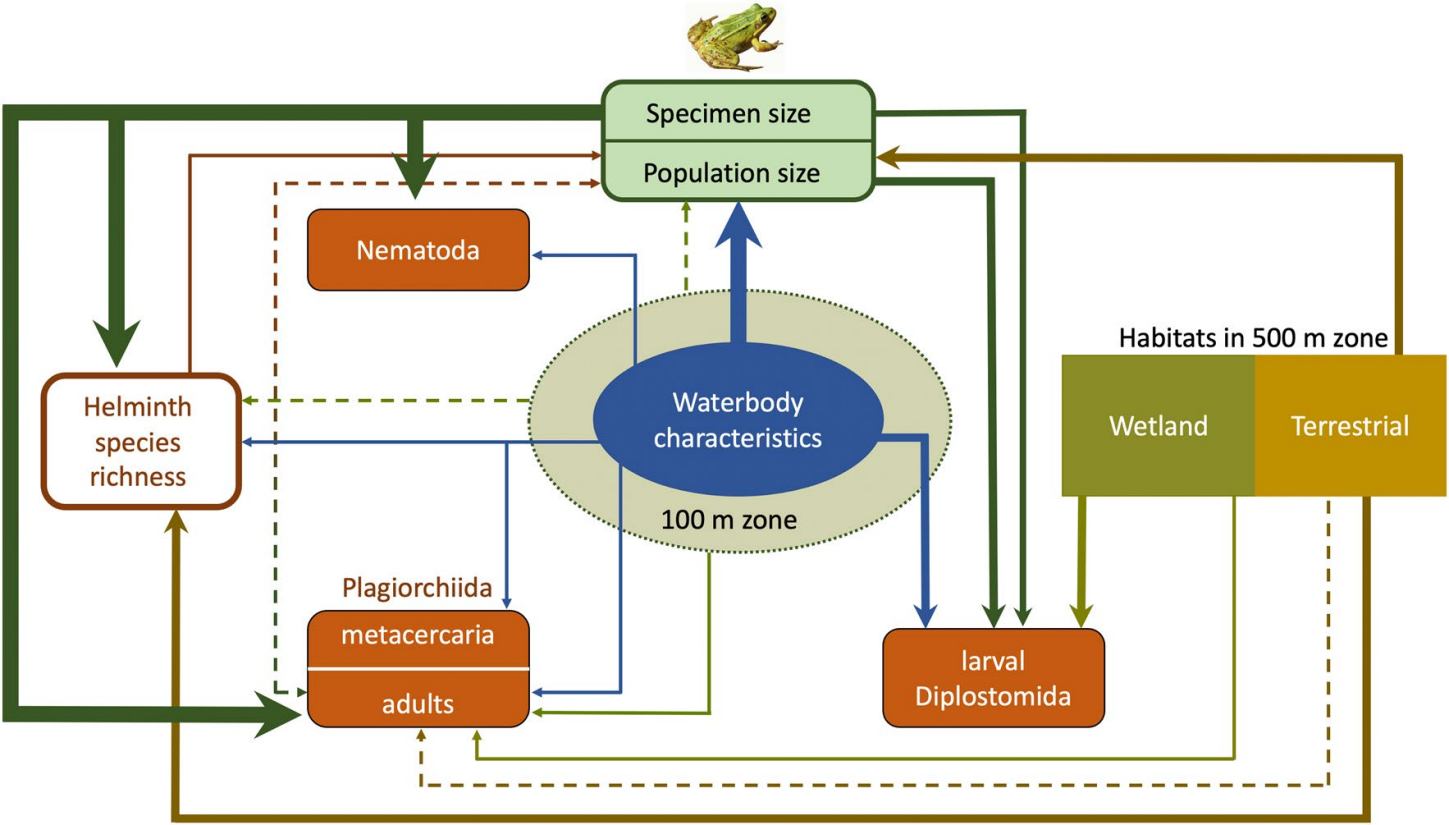
Flow chart showing relationships between the water frog-helminth-environment system components in our study. The thickness and continuity of arrow-headed lines is proportional to the strength of their suggested effects; helminth species richness refers to the infra-community richness (species richness in individual frog hosts), but different helminth groups refer to their abundances in hosts.

Helminth groups and their development stages notably varied in responses to ecological factors, both in the range of factors and in the strengths of their effects (Fig. 3). Thus, abundances of plagiorchiid trematode adult stages had relatively weak responses for many environmental factors that would be expected to affect both parasite and host populations^39,40^. Environmental effect was almost non-detectable in plagiorchiid larvae, while diplostomid larvae had relatively strong responses to several predictors (Fig. 2), which could be attributed to same factor—availability of permanent shallow waterbodies. This pattern could be explained by the differences in life cycles between these trematode groups. Diplostomid trematodes generally have a free-swimming miracidium stage which results in poorer tolerance to fluctuations of environmental conditions (not only in water levels but also water chemistry, densities of micropredators etc.) compared to most of plagiorchiid trematodes, whose miracidia hatch from the eggs in the intestines of the mollusc^41^.

Many indirect effects of environmental factors through host populations should be present, and some of them could be suggested by our results: (i) plagiorchiid adult stages showed higher abundances in areas with few waterbodies and mires that could be caused by intermediate (arthropod) and definitive (frogs) host attraction form a larger area and higher helminth transmission rates; (ii) in larval diplostomids, positive response to waterbody area, and negative responses to agriculture and human settlements corresponded to the same but stronger effects of these factors on water frog population size (Tables 2 and 3, Fig. 2), suggesting that they are actually host-mediated indirect effects on diplostomid abundances.

### Mutual dependence of frog and helminth populations

Relationships between components of the water frog-helminth-environment system in our study suggests that the development of human settlements may cause a three-step parasite community shift through (i) increasing of helminth species richness (most likely associated with populations of domestic animals^42^), which may (ii) depress the host (water frog) population, which in turn (iii) has adverse effects on those parasite groups that benefits from high host densities. Taking into account the top-down effect assessment (see above), it can be inferred that the helminths typically have little effect on the water frog population size, except for the cases when hosts get overloaded with too many helminth species for host fitness and survival^33^ that in turn may depress helminth infra-communities through temporary or permanent reduction of the host population size, when decrease in the host population density may drop infection rates^43,44^. Hence, the mutual dependence of population sizes in a given parasite-host system helps to keep it in a balance without over-exploiting host populations^45^. In the systems we studied such a mechanism primarily regulates diplostomid infections, which are common and probably the most harmful infra-community component in the water frogs^9,17^. Such a balance can be shifted to a new state by an environmental trigger that can be an anthropogenic impact, like the human settlements in our study, or any other ecological factor, such as predator populations^46,47^, thus keeping this parasite-host system in a state of dynamic equilibrium.

### Conclusions, limitations, and further directions

Our study identifies the presence of not only top-down but also bottom-up effects in the water frog–helminth system at the population level and offers the description of a mechanism, which facilitates sustainable exploitation of the amphibian host resource by helminths.

It should be noted that in our study we had a relatively small number of sampled frogs in most of the sites, what may cause substantial random deviations from the true values in our helminth abundance and species richness estimations. In addition, we performed purely correlational research, where causes of correlations may be interpreted erroneously. To overcome these limitations, in our study we focused on general relationships and supported our interpretations with the results from the other studies. However, in order to confirm our results, experimental research with manipulation of the frog population size is recommended.

Our study also demonstrated good potential for the use of the vocalizing amphibian male counts for the estimation of amphibian population relative size. Calling male counts have shown themselves as a resource-efficient tool that can be applied in correlational research or field experiment studies, both in fields of parasitology and amphibian ecology. Our explanation on how the water frog populations maintain in balance with helminth infections could still be oversimplified, and wider correlational research-type studies with larger datasets, more environmental variables and inclusion of population size estimates not only for amphibians, but also for trophic levels placed both beneath (snails and invertebrate prey) and above (amphibian predators) amphibians are welcomed.

## Supplementary Information

Supplementary Information.

## Data Availability

The original data of the authors used in this study is available in the Supplement 1 of the present paper.
