# Supplementary material for: Top-down and bottom-up effects and relationships with local environmental factors in the water frog–helminth systems in Latvia
Source: Sci Rep. 2023 May 27;13:8621. doi: 10.1038/s41598-023-35780-7 (PMC10224975; doi:10.1038/s41598-023-35780-7)
Supplement: Supplementary file 1 — Supplementary Information. [file 41598_2023_35780_MOESM1_ESM.pdf]

Supplement 1

The full data set analysed in Čeirāns *et al* . "Top-down and bottom-up effects and relationships with local environmental factors in the water frog–helminth systems in Latvia"

				Host (H) variables		*Parasite (P) variables*					*Waterbody (W) variables*								
Site	Latitude N	Longitude E	Parasitology samples	Host size (cm)	Calling frogs	Diplostomid larv (A)	Plagiorchiid larv (A)	Plagiorchiid ad (A)	Nematoda larv (A)	Species richness (S)	Area (ha)	Permanence	Depth (m)	Bottoms (category)	Submersed vegetation (%)	Floating vegetation (%)	Emergent vegetetion (%)	Shore woods	Shore reeds
Abava	57.102447°	22.024804°	5	6,0	7,5	0,0	0,0	0,8	0,0	0,4	2,28	0,42	1,2	3	90	20	60	0,16	0,00
Ainavas	55.834767°	26.484259°	3	5,8	3,5	1,0	1,7	15,3	0,3	5,3	0,08	0,74	1,5	1	40	10	15	0,38	0,00
Bikaunieki1	56.383364°	27.176981°	5	3,5	7,5	0,0	1,8	2,0	0,0	2,0	0,06	0,22	0,7	2	100	20	80	0,74	0,00
Bikaunieki2	56.379359°	27.195667°	3	3,9	7,5	0,0	0,0	0,0	0,0	0,0	0,38	0,43	1,0	3	90	20	20	0,40	0,00
Cesis	57.328508°	25.297681°	3	7,0	2,5	0,0	0,0	18,0	7,0	2,0	0,02	0,91	1,0	3	100	90	20	0,44	0,00
Dinsdurbe1	56.539057°	21.718932°	6	4,8	7,5	8,3	0,0	0,7	0,2	1,2	0,13	0,29	1,2	3	90	50	40	0,00	0,00
Dinsdurbe2	56.540786°	21.712050°	5	5,4	7,5	3,4	0,0	0,0	0,0	1,2	1,06	0,73	5,0	3	40	10	20	0,82	0,00
Dviete1	56.069937°	26.225928°	14	6,9	4,5	0,9	0,7	4,9	1,0	2,4	0,06	0,30	0,7	3	0	0	20	0,00	0,00
Dviete2	56.046484°	26.279376°	3	6,7	3,5	8,7	0,0	0,3	0,0	1,3	0,10	0,79	1,5	1	50	5	5	0,25	0,00
Dviete3	56.044664°	26.278425°	4	7,3	1,0	0,3	1,8	2,0	0,0	1,0	0,13	0,54	1,5	1	50	5	10	0,39	0,00
Eglaine1	55.959649°	26.061459°	4	5,9	1,0	3,0	0,0	2,5	0,5	3,0	0,62	0,75	1,5	3	40	5	20	0,50	0,36
GNP_T4	57.219169°	25.110288°	4	2,8	15,5	0,0	0,3	6,8	0,0	0,5	0,93	0,68	1,2	3	5	80	30	0,79	0,00
GNP_T11	57.240246°	25.206915°	4	4,0	5,0	0,0	7,0	22,5	0,0	1,5	0,10	0,36	1,0	3	100	60	40	0,29	0,00
GNP_T19	57.329136°	25.298477°	3	2,7	4,0	0,0	0,0	0,0	0,0	0,0	0,11	0,67	1,0	3	80	80	20	0,60	0,00
GNP_T33	57.363316°	25.305229°	3	2,3	7,5	0,0	2,0	0,0	0,0	2,0	0,42	0,39	1,0	3	100	20	60	0,54	0,00
GNP_T37	57.279745°	25.391975°	3	3,5	5,5	0,0	0,0	0,0	0,0	0,0	1,50	0,85	1,0	3	20	20	20	0,39	0,41
GNP_T49	57.315509°	25.221045°	3	5,8	3,5	10,0	0,0	4,0	6,0	6,0	0,63	0,03	0,5	3	10	20	30	0,00	0,00
GNP_T52	57.318837°	25.197195°	3	3,0	1,0	0,0	0,0	0,0	0,0	0,0	0,03	0,57	1,0	2	20	20	20	0,08	0,00
GNP_T55	57.308605°	25.163995°	4	3,0	7,5	52,8	0,5	0,0	0,0	1,3	0,27	0,13	1,5	2	20	20	30	0,00	0,00
GNP_T63	57.302239°	25.041052°	3	6,6	1,0	0,0	11,0	1,0	0,0	2,0	0,04	0,60	0,7	1	10	10	20	0,00	0,00
GNP_T68a	57.304007°	24.973356°	3	2,0	2,5	0,0	0,0	0,0	0,0	0,0	0,05	0,10	0,2	3	0	0	90	0,00	0,00
GNP_T78	57.234474°	25.052245°	3	3,4	4,5	0,0	0,0	0,0	0,0	0,0	0,02	0,08	0,5	3	80	30	80	0,33	0,00
GNP_T79	57.247760°	25.014531°	6	3,0	4,0	0,0	0,3	0,0	0,0	0,2	0,05	0,33	0,7	3	80	60	40	0,47	0,00
GNP_T84	57.173178°	25.034701°	3	2,5	4,5	0,0	1,0	0,0	0,0	1,0	1,01	0,02	0,5	3	60	20	60	0,26	0,00
GNP_T91	57.103496°	25.096269°	3	5,0	1,0	0,0	1,0	2,0	0,0	2,0	0,02	0,19	0,7	3	80	80	20	0,00	0,00
GNP_T95a	57.157295°	24.838851°	3	2,3	1,0	9,0	9,0	0,0	1,0	4,0	0,07	0,05	0,2	3	0	100	2	0,00	0,49
GNP_T105	57.215524°	24.816406°	3	2,9	3,5	0,0	0,0	0,0	0,0	0,0	0,18	0,11	1,0	2	80	80	20	0,72	0,00
GNP_T110	57.175524°	24.769301°	6	2,9	1,0	0,2	6,2	4,5	0,2	1,5	0,06	0,57	1,0	3	10	10	20	0,04	0,21
GNP_T113	57.168578°	24.719128°	4	4,0	13,5	29,0	0,0	0,0	0,5	1,0	1,45	0,61	1,0	2	80	20	30	0,38	0,00
Ilgas1	55.692886°	26.787292°	27	5,5	12,5	7,3	10,7	20,7	0,4	3,0	0,08	0,52	0,9	2	80	20	80	0,52	0,00
Ilgas2	55.690944°	26.771224°	9	5,3	8,0	4,8	0,0	0,4	0,0	0,4	0,03	0,13	0,7	3	90	90	20	0,48	0,00
Ilgas3	55.690767°	26.788733°	4	5,1	7,0	37,3	2,3	2,8	0,0	3,0	0,01	0,76	0,8	3	30	10	20	0,07	0,00
Ilgas4	55,690973	26,789023	9	2,7	7,5	1,8	7,1	0,3	0,0	1,1	0,02	0,05	0,8	3	30	10	20	0,03	0,00
Jelgava1	56.714404°	23.680851°	3	5,7	4,0	0,0	0,0	6,0	0,0	1,3	0,35	0,51	1,5	1	80	1	5	0,09	0,18
Jelgava2	56.713403°	23.680483°	3	6,6	5,0	1,7	3,7	1,3	0,0	1,7	0,05	0,50	1,0	1	80	5	1	0,02	0,00
KNP_T2	56.842843°	23.400174°	3	6,5	3,5	0,0	4,0	33,0	0,0	2,0	0,05	0,77	0,7	1	40	30	20	0,00	0,00
KNP_T6	56.821577°	23.474087°	4	4,8	5,0	0,0	0,0	3,0	0,0	1,0	0,43	0,79	1,0	2	40	30	60	0,22	0,57
KNP_T7	56.819555°	23.477318°	4	5,5	4,0	0,0	0,0	2,5	0,0	2,0	1,12	0,55	0,5	2	60	20	80	0,43	0,38
KNP_T8	56.824938°	23.469954°	3	4,0	2,0	0,0	0,0	0,0	0,0	0,0	0,32	0,81	0,7	2	60	20	80	0,17	0,83
Kraslava1	55.932730°	27.209319°	5	7,2	4,5	53,4	2,4	2,4	5,2	4,2	0,02	0,55	0,5	3	0	100	5	0,90	0,00
Kraslava3	55.947721°	27.227083°	3	5,6	3,5	44,0	0,0	6,0	0,0	2,0	0,37	0,42	1,0	3	80	50	10	0,03	0,00
Krivanda1	56.504178°	28.035008°	3	7,7	3,0	0,0	1,0	24,0	3,0	5,0	0,04	0,94	1,0	1	60	20	30	0,26	0,00
Krivanda2	56.541140°	28.061643°	3	3,5	1,0	0,0	0,0	0,0	0,0	0,0	0,01	0,77	1,0	1	50	20	40	0,00	0,00
Kuldiga1	57.006246°	22.014219°	3	6,9	5,0	0,0	0,0	0,0	0,0	0,0	0,08	0,46	0,7	3	30	10	1	0,20	0,00
Kuldiga2	57.012402°	22.049936°	3	5,7	2,0	0,0	0,0	0,3	0,0	0,3	0,01	0,20	0,5	3	0	0	100	0,10	0,00
Lubans_T22b;	57.005229°	26.872249°	5	6,6	3,5	9,2	3,8	9,2	0,0	3,6	0,08	0,51	1,0	2	100	2	2	0,94	0,00
Lubans_T35	57.053427°	26.878515°	4	6,2	3,0	1,5	8,3	5,8	0,0	2,0	0,02	0,08	0,7	1	60	5	10	0,12	0,00
Lubans_T38	57.051209°	26.886105°	4	4,7	1,0	0,0	3,0	5,5	3,0	4,0	0,03	0,36	1,0	2	20	100	10	0,97	0,00
Lubans_T39	57.050659°	26.887904°	4	5,3	1,5	0,0	0,0	7,5	0,0	0,5	0,01	0,53	0,5	3	100	80	30	0,45	0,00
Lubans38A	56.839690°	26.866000°	3	5,5	7,0	59,0	0,0	0,0	7,0	3,0	0,05	0,84	0,7	3	0	100	5	0,72	0,00
Lubans51B	56.752610°	26.949920°	3	6,7	7,5	0,0	0,0	48,5	9,0	5,0	0,07	0,82	1,0	3	20	10	20	0,05	0,00
Lubans51C	56.751520°	26.948730°	3	5,2	8,0	3,7	0,0	6,3	4,3	3,3	0,14	0,44	1,0	3	30	10	20	0,39	0,00
Malta	56.340703°	27.153661°	7	3,1	6,0	0,7	0,0	0,0	0,0	0,1	0,01	0,78	0,7	3	80	20	20	0,10	0,00
Saldus2	56.601554°	22.312442°	3	5,3	7,5	5,0	0,0	5,0	1,3	2,0	0,19	0,41	0,5	3	100	90	30	0,88	0,00
Saldus3	56.620572°	22.287358°	5	4,9	3,5	3,4	0,0	1,8	0,0	1,0	0,01	0,50	1,2	3	80	10	20	0,12	0,00
Saldus4	56.605332°	22.326657°	6	5,1	4,5	24,2	0,0	0,2	0,0	0,3	0,03	0,24	0,5	3	95	20	30	0,00	0,00
Saldus5	56.583117°	22.272965°	6	4,4	7,5	10,5	0,0	0,3	0,0	1,0	0,13	0,13	0,3	3	10	5	10	0,50	0,00
Sigulda	57.165717°	24.852636°	3	6,0	2,5	4,0	9,0	58,0	2,0	8,0	0,29	0,31	1,2	3	30	100	20	0,78	0,00
Silene	55.721551°	26.708519°	3	6,5	1,0	5,0	0,0	3,0	0,0	3,0	0,01	0,38	0,7	3	95	95	20	0,83	0,00
Tukums_T10	57.007919°	23.176134°	4	4,4	3,5	0,0	1,0	1,0	0,5	2,0	0,03	0,75	1,0	3	20	20	30	0,00	0,00
Tukums_T11	57.014025°	23.212365°	3	5,3	2,5	0,0	0,0	2,0	0,0	3,0	0,01	0,51	1,0	3	10	100	30	0,07	0,00
Tukums_T13	57.003263°	23.205439°	3	7,4	2,5	0,0	0,0	4,0	0,0	2,0	0,08	0,76	1,0	1	40	30	10	0,15	0,23
Vaide	57.726631°	22.472377°	3	6,0	4,5	17,0	0,0	0,0	1,0	1,0	0,57	0,43	1,2	3	60	10	60	0,55	0,00

	Landuse within 100 m (L100) variables					*Landuse within 500 m (L500) variables*				
Site	forest100	mires100	agriculture 100	settlements 100	waters100	forest500	mires500	agriculture 500	settlement s500	waters500
Abava	0,462	0,454	0,000	0,000	0,022	0,641	0,307	0,000	0,000	0,028
Ainavas	0,050	0,000	0,335	0,105	0,020	0,210	0,025	0,261	0,078	0,022
Bikaunieki1	0,463	0,042	0,140	0,000	0,054	0,420	0,008	0,168	0,001	0,001
Bikaunieki2	0,082	0,061	0,432	0,018	0,006	0,187	0,009	0,588	0,004	0,004
Cesis	0,462	0,000	0,045	0,019	0,032	0,403	0,007	0,209	0,070	0,008
Dinsdurbe1	0,158	0,298	0,000	0,000	0,044	0,421	0,175	0,000	0,000	0,047
Dinsdurbe2	0,669	0,030	0,000	0,000	0,000	0,655	0,072	0,000	0,000	0,051
Dviete1	0,001	0,029	0,963	0,000	0,000	0,134	0,007	0,828	0,003	0,001
Dviete2	0,315	0,007	0,277	0,000	0,000	0,374	0,013	0,403	0,003	0,002
Dviete3	0,532	0,004	0,144	0,000	0,002	0,425	0,013	0,310	0,000	0,001
Eglaine1	0,348	0,005	0,433	0,000	0,035	0,377	0,000	0,462	0,010	0,005
GNP_T4	0,579	0,041	0,120	0,000	0,078	0,482	0,037	0,327	0,003	0,023
GNP_T11	0,637	0,000	0,000	0,004	0,000	0,613	0,003	0,120	0,018	0,015
GNP_T19	0,547	0,017	0,008	0,036	0,034	0,429	0,006	0,194	0,068	0,007
GNP_T33	0,800	0,015	0,000	0,000	0,053	0,758	0,003	0,009	0,002	0,140
GNP_T37	0,344	0,022	0,280	0,000	0,002	0,224	0,002	0,603	0,006	0,001
GNP_T49	0,459	0,039	0,445	0,000	0,005	0,687	0,013	0,045	0,007	0,108
GNP_T52	0,223	0,000	0,538	0,000	0,000	0,268	0,003	0,482	0,027	0,005
GNP_T55	0,392	0,011	0,145	0,035	0,000	0,407	0,031	0,044	0,037	0,279
GNP_T63	0,241	0,000	0,573	0,000	0,000	0,615	0,071	0,255	0,000	0,002
GNP_T68a	0,664	0,104	0,000	0,000	0,000	0,745	0,058	0,003	0,000	0,006
GNP_T78	0,208	0,000	0,006	0,302	0,000	0,503	0,000	0,025	0,110	0,006
GNP_T79	0,686	0,000	0,058	0,011	0,000	0,802	0,006	0,078	0,005	0,050
GNP_T84	0,292	0,148	0,125	0,022	0,000	0,383	0,008	0,101	0,080	0,004
GNP_T91	0,496	0,017	0,142	0,031	0,006	0,362	0,025	0,281	0,044	0,011
GNP_T95a	0,537	0,142	0,000	0,005	0,037	0,560	0,017	0,000	0,096	0,074
GNP_T105	0,706	0,000	0,000	0,007	0,040	0,553	0,008	0,143	0,005	0,058
GNP_T110	0,028	0,003	0,775	0,008	0,019	0,173	0,001	0,703	0,014	0,002
GNP_T113	0,540	0,013	0,225	0,001	0,002	0,377	0,001	0,397	0,013	0,001
Ilgas1	0,411	0,057	0,000	0,000	0,005	0,610	0,009	0,009	0,005	0,005
Ilgas2	0,721	0,049	0,000	0,000	0,011	0,881	0,032	0,000	0,000	0,003
Ilgas3	0,403	0,122	0,046	0,000	0,019	0,624	0,054	0,017	0,008	0,005
Ilgas4	0,514	0,141	0,006	0,000	0,017	0,626	0,053	0,016	0,008	0,005
Jelgava1	0,237	0,000	0,017	0,000	0,015	0,338	0,000	0,396	0,022	0,017
Jelgava2	0,111	0,000	0,135	0,000	0,056	0,337	0,000	0,394	0,022	0,021
KNP_T2	0,018	0,000	0,766	0,003	0,047	0,570	0,000	0,133	0,000	0,002
KNP_T6	0,838	0,153	0,000	0,000	0,000	0,908	0,023	0,000	0,000	0,026
KNP_T7	0,655	0,330	0,000	0,000	0,000	0,913	0,027	0,000	0,000	0,021
KNP_T8	0,912	0,088	0,000	0,000	0,000	0,954	0,007	0,000	0,000	0,021
Kraslava1	0,444	0,000	0,000	0,003	0,019	0,591	0,026	0,083	0,001	0,124
Kraslava3	0,248	0,055	0,472	0,003	0,000	0,406	0,041	0,438	0,003	0,006
Krivanda1	0,807	0,000	0,000	0,000	0,000	0,829	0,001	0,000	0,000	0,001
Krivanda2	0,648	0,000	0,000	0,000	0,000	0,769	0,000	0,046	0,008	0,001
Kuldiga1	0,389	0,000	0,396	0,000	0,022	0,474	0,001	0,202	0,005	0,092
Kuldiga2	0,614	0,000	0,000	0,000	0,000	0,777	0,018	0,000	0,000	0,001
Lubans_T22b;	0,765	0,004	0,000	0,000	0,061	0,722	0,000	0,089	0,005	0,072
Lubans_T35	0,439	0,031	0,000	0,070	0,123	0,618	0,041	0,114	0,004	0,067
Lubans_T38	0,398	0,000	0,380	0,000	0,109	0,657	0,011	0,133	0,003	0,051
Lubans_T39	0,551	0,011	0,266	0,000	0,017	0,690	0,010	0,132	0,003	0,043
Lubans38A	0,152	0,000	0,408	0,000	0,056	0,317	0,019	0,270	0,004	0,017
Lubans51B	0,166	0,000	0,549	0,024	0,019	0,099	0,053	0,318	0,014	0,016
Lubans51C	0,212	0,000	0,395	0,066	0,022	0,097	0,040	0,392	0,012	0,016
Malta	0,108	0,000	0,252	0,116	0,014	0,306	0,001	0,248	0,111	0,007
Saldus2	0,824	0,135	0,000	0,000	0,000	0,594	0,098	0,176	0,000	0,006
Saldus3	0,857	0,013	0,000	0,000	0,006	0,907	0,001	0,000	0,000	0,001
Saldus4	0,420	0,022	0,125	0,000	0,029	0,465	0,055	0,203	0,002	0,081
Saldus5	0,678	0,214	0,000	0,000	0,000	0,622	0,042	0,137	0,000	0,002
Sigulda	0,465	0,003	0,000	0,126	0,000	0,671	0,000	0,000	0,119	0,003
Silene	0,580	0,256	0,000	0,000	0,000	0,737	0,103	0,028	0,000	0,008
Tukums_T10	0,475	0,014	0,022	0,047	0,022	0,546	0,038	0,094	0,022	0,056
Tukums_T11	0,357	0,020	0,000	0,000	0,000	0,751	0,088	0,007	0,003	0,007
Tukums_T13	0,288	0,000	0,403	0,000	0,033	0,622	0,010	0,151	0,012	0,055
Vaide	0,832	0,081	0,000	0,000	0,087	0,888	0,052	0,000	0,005	0,036
